# Optical surgical navigation-assisted removal of a foreign body using a splint to simplify the registration process: a case report

**DOI:** 10.1186/s13256-019-2159-8

**Published:** 2019-07-09

**Authors:** Shintaro Sukegawa, Shingo Yoneda, Takahiro Kanno, Hidetoshi Tohmori, Yoshihiko Furuki

**Affiliations:** 1 Division of Oral and Maxillofacial Surgery, Kagawa Prefectural Central Hospital, 1-2-1, Asahi-machi, Takamatsu, Kagawa 760-8557 Japan; 2 Department of Oral and Maxillofacial Surgery, Federation of National Public Services and Affiliated Personnel Mutual Aid Associations, Kure Kyosai Hospital, Hiroshima, Japan; 3 Department of Oral and Maxillofacial Surgery, Shimane University Faculty of Medicine, Shimane, Japan

**Keywords:** Navigation system, Foreign bodies, Splint, Registration, Maxilla

## Abstract

**Background:**

Oral and maxillofacial surgeons often encounter foreign objects within the human body. Despite the visual identification of foreign objects via imaging techniques, the accurate determination of their position in the maxillofacial area can be challenging. The clinical application of a navigation system can solve this issue. This system provides a useful guide for a safer and more accurate surgical technique by accurately determining the location of the lesion in real time during the surgery. However, complications with regard to registration may be encountered. We describe a navigation system that simplifies registration using a dental splint with embedded reference points for foreign body removal in the maxilla.

**Case presentation:**

A 78-year-old Japanese woman was referred with the chief complaint of pain in the left upper molar region. We found the symptoms to be associated with a foreign body in the maxilla and decided to remove it. A minimally invasive treatment procedure was desirable. However, the lesion was in contact with the maxillary sinus, and it was difficult to pinpoint its position because of the absence of an anatomical landmark. Therefore, we decided to use a navigation system. In order to simplify registration, a dental splint with embedded reference points was created. The registration could be reliably performed before surgery using an optical navigation system that facilitates the process, using splints with embedded reference points. Following preoperative registration, the splint with the reference frame was placed in the patient’s mouth, and the accuracy of the navigation was confirmed. The position with respect to the maxillary sinus was precisely identified followed by the removal of the surrounding bone and excision of the lesion. Therefore, the surgery could be accurately performed without perforating the maxillary sinus. In addition, owing to preoperative registration, the operative time could be shortened. After the surgical procedure, the patient’s symptoms disappeared.

**Conclusions:**

The procedure was performed in a precise, minimally invasive manner. Furthermore, the operative time was reduced by the simplified registration process, wherein a splint was embedded with reference points. This technique may prove useful for performing maxillofacial surgical procedures.

## Background

Foreign bodies are defined as objects originating outside the human body. The most commonly retained foreign objects in the body are wood splinters, glass fragments, and metal substances [1]. They are generally found to occur as a result of traffic accidents, assaults, bullet wounds, and iatrogenic dental injuries [2]. Some of the foreign objects retained in the body during dental treatment include canal sealers, amalgam, cement, mineral trioxide aggregate, and Ca(OH)_2_. Radiographs, computed tomograms (CT), magnetic resonance images, and ultrasound may be used to locate these foreign objects, depending on their location and composition [3, 4]. However, despite the visual identification of a foreign body via imaging, the accurate determination of its position within the tissues in the maxillofacial area can be challenging.

Computer-assisted navigation systems have recently evolved to simplify and improve the precision of the surgical procedure to minimize surgical invasiveness. Therefore, the execution and predictability of surgical procedures has improved, allowing for more precision during maxillofacial surgery [5]. Complications such as registration and deterioration of accuracy are important issues during intraoperative navigation.

We describe a navigation system that simplifies registration using a dental splint with embedded reference points for the removal of a foreign body in the maxilla.

## Case Presentation

**Fig. 1:**
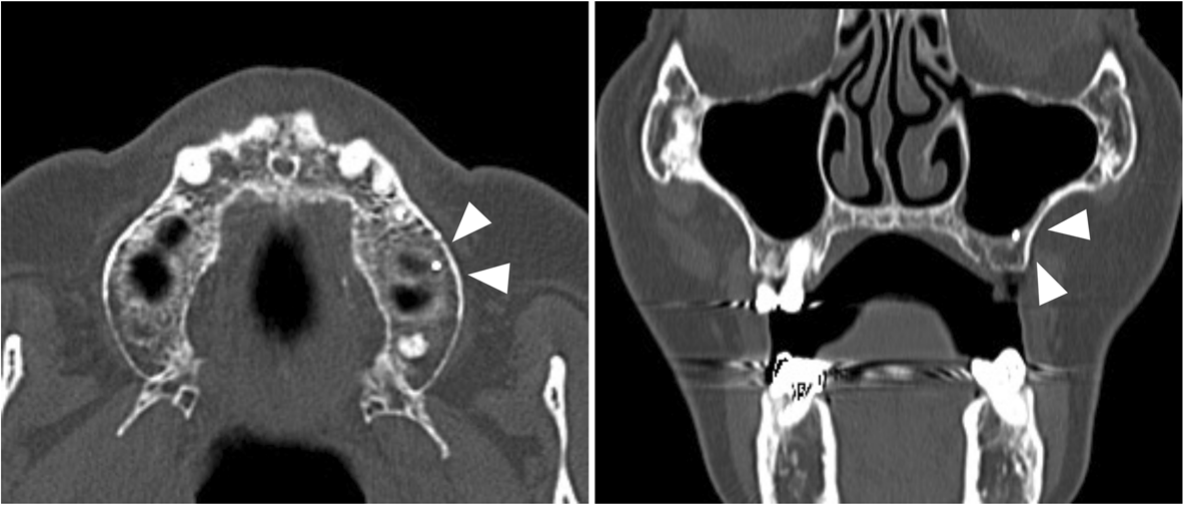
The size of the radiopaque lesion in the CT image was 2 mm diameter (arrow), and the lesion was located in the maxillary bone partly in contact with the base of the maxillary sinus

A 78-year-old Japanese woman was referred to the Division of Oral and Maxillofacial Surgery at the Kagawa Prefectural Central Hospital in 2017 with the chief complaint of constant pain in the left upper molar region. She had undergone left maxillary first molar extraction 10 years ago, but details on root canal treatment were unavailable. Her discomfort did not disappear even after tooth extraction, and recently, the pain had been exacerbating. Panoramic radiographs revealed a radiopaque lesion in contact with the maxillary sinus at the apical portion of a missing left maxillary first molar. The size of the radiopaque lesion in the CT image was 2 mm diameter, and it was located in the maxillary bone partly in contact with the base of the maxillary sinus (Fig. 1). It was perfectly consistent with the part associated with the patient’s pain complaint. We diagnosed the symptoms as being caused by the foreign body in the maxilla and decided to remove the object. A minimally invasive treatment procedure was desirable; however, the lesion was in contact with the maxillary sinus, and it was difficult to pinpoint its position because of the absence of an anatomical landmark, such as a tooth or a fossa. Therefore, we decided to apply a surgical navigation system to locate and remove the object.

### Technique

#### Preparing the registration splint

**Fig. 2:**
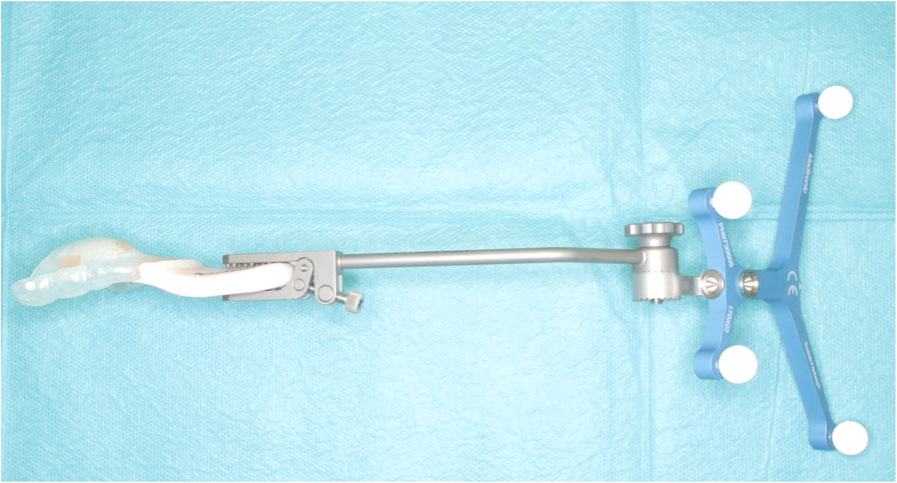
A navigational splint with a reference frame

A custom splint was fabricated with acrylic resin using a dental mold. To fix the reference frame and arrange the points for registration more stereoscopically, the splint was extended. In total, ten temporary stopping dental markers were incorporated for marker-based pair-point registration (Fig. 2).

#### Preparation for navigation

**Fig. 3:**
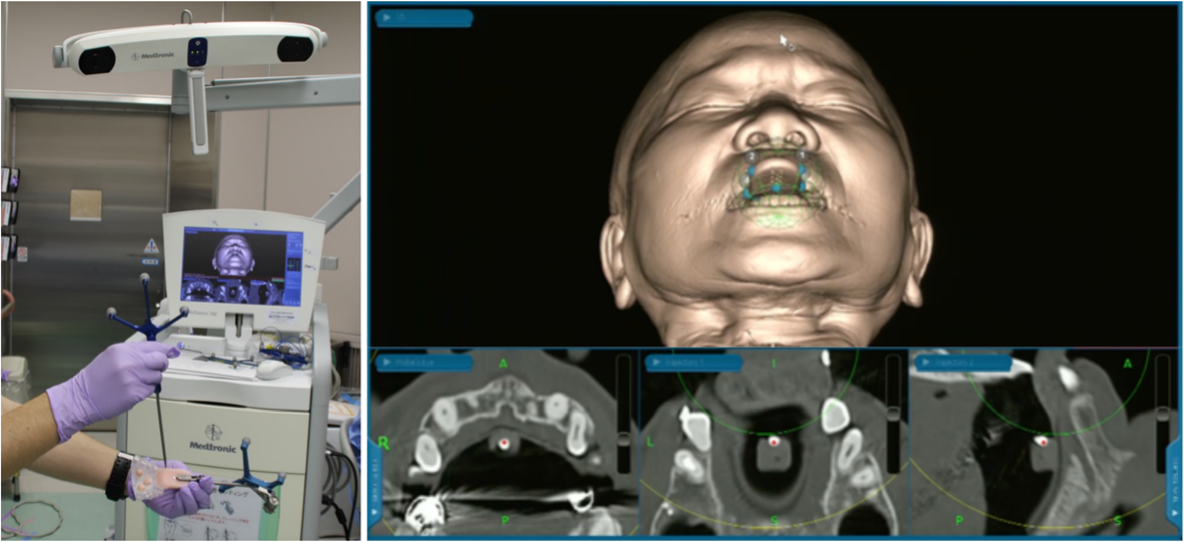
Preoperative registration is performed with a splint and a fixed reference frame; it can be performed in the absence of the patient

CTs of the region with the attached splint were obtained using the DICOM (Digital Imaging and Communication in Medicine) format and transferred to a Medtronic StealthStation S7 workstation, which used the Synergy Fusion Cranial 2.2.6 software (Medtronic Navigation Inc., Louisville, CO, United States). Registration was performed before the surgical procedure. After the reference frame was attached to the splint, point registration was performed using the point embedded in the splint. This procedure can be performed without a patient because preoperative registration is performed only with a splint that is fixed to a reference frame (Fig. 3).

#### Surgical procedure

**Fig. 4:**
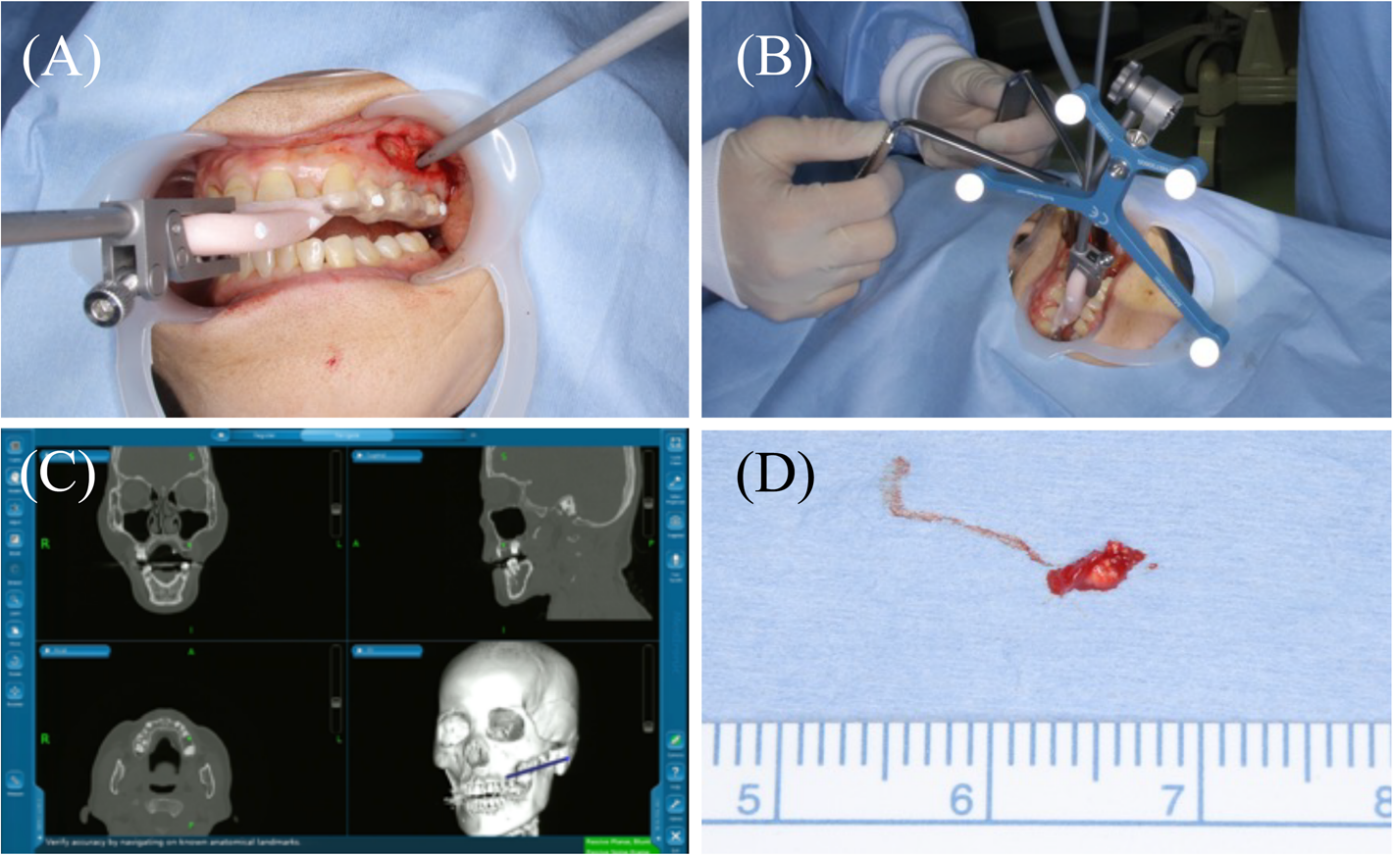
**a, b** Intraoperative view. **c** The splint was attached along with a reference frame, and the location of the lesion was identified using the navigation system. **d** The foreign body was removed

Following preoperative registration, the splint with the reference frame was placed in the patient’s mouth, and the accuracy of the navigation was confirmed. The mucosal periosteum was detached to expose the maxillary bone, and the position of the lesion in the maxilla was identified by navigation (Fig. 4). The position with regard to the maxillary sinus was identified followed by the removal of the surrounding bone and excision of the lesion. Therefore, the surgery could be accurately performed without perforating the maxillary sinus. After the surgical procedure, the patient’s symptoms disappeared.

## Discussion

Oral and maxillofacial surgeons often encounter foreign bodies within the dentomaxillofacial and oral tissues [6]. Foreign bodies in the maxillofacial area occur as a sequela of trauma or therapeutic interventions. Among them, the most frequent foreign bodies are dental filling materials within the jawbones [7]. Foreign bodies can lead to significant complications such as irritation, inflammation, infection, abscess formation, pain, swelling, and potential injury to surrounding vessels or nerves [8]. In some asymptomatic cases, the objects may be retained within the tissues, depending on the location and characteristics of the foreign body. In other cases, foreign bodies should be removed in the presence of clinical symptoms.

Although various imaging modalities are selected on the basis of size, composition, and anatomic relation of the foreign bodies to different vital structures [9], CT imaging is the gold standard for the detection of foreign bodies [8, 10]. In recent years, CT imaging has become very easy owing to the introduction of cone beam CT. The presence of an object can be confirmed using these imaging techniques. Yet, an accurate determination of the position of a foreign body in the maxillofacial region can be challenging in some cases. This may be attributed to the small size of the foreign body and the limited inflammatory response in some instances. Furthermore, if the object is not adjacent to immobile anatomical landmarks, it is extremely difficult to determine its position accurately [11]. Furthermore, it is necessary to accurately determine the spatial position of the object with regard to important organs in the vicinity.

Clinical reports have demonstrated various surgical strategies for navigation-guided surgery during maxillofacial treatment [12, 13]. Navigation systems are helpful in identifying the location of the foreign body, determining the optimal approach, and performing the surgical procedure using a minimum surgical invasion strategy [14–16]. However, there are many problems to be considered with regard to the use of the navigation system.

First, the operative time is extended due to procedures such as navigation setup and registration. According to previous reports, it took approximately 15–20 minutes to prepare the area for navigation [16–18]. An accurate registration process is essential for successful navigation. If there is an error in registration, it has to be repeated several times to achieve an accurate registration. The technique used in the present report can perform the registration process preoperatively via optical navigation. Electromagnetic navigation systems use electromagnetic fields that employ reference points on a device attached to the patient’s head, such as a plastic mask with metallic beads or headband and a wired instrument that the surgeon uses within the maxillofacial region. Therefore, registration cannot be performed without a patient in electromagnetic navigation. Conversely, in optical navigation, point registration can be performed without a patient by setting a pointer with a marker on the splint before surgery. Therefore, registration can be performed before the patient enters the operating room and does not affect the operative time. In other words, it is expected to considerably shorten the operative time. In cases in which the surgeon is unfamiliar with the navigation system, the surgical procedure can be performed reliably without worrying about the operative time.

Second, the range of CT images during navigation is generally wide. Registration techniques can be categorized into two main groups: marker-based and marker-free [5]. Marker-free registration relies on the patient’s craniofacial anatomy. Therefore, for accurate registration, it is necessary to have a part of the face unrelated to the lesion. As a result, the range of CT imaging is widened, and exposure to radiation is increased. The technique used in the present study was marker-based, where the reference marker is embedded in the splint; therefore, only a CT range involving the lesion and the splint is sufficient.

Third, the accuracy of registration needs to be maintained during surgical navigation. In the case of marker-free registration, deterioration of navigation accuracy has been noted in response to changes in soft tissues during surgery [19]. Similarly, there is a reduction in accuracy owing to a slight change in the position of the registration frame due to a change in head position during maxillofacial surgery. The advantage of the technique used in this study is that the positional relationship between the marker used for registration and the reference frame is unchanged. Therefore, it is easy to maintain navigation accuracy. In the present study, re-registration was not performed during surgery.

In the future, this technique can be used for navigation in the mandible because the positional relationship between the marker used for registration and the reference frame remains unchanged. Various studies have reported mobility as a limitation for the use of surgical navigation in the mandible [20, 21]. The technique described in the present report may be used for various treatments in the maxillofacial area in the future.

## Conclusions

We report a case of a patient from whom a foreign body in the maxillary bone was removed using surgical navigation. The procedure was performed in a precise, minimally invasive, and safe manner. Furthermore, the operative time was reduced by the simplified registration process, wherein a splint was embedded with a reference point. We believe that this technique may prove useful when performing maxillofacial surgical procedures.

## Data Availability

All data generated or analyzed during this study are included in this published article.
